# Bloodless Surgery of the Extremities

**Published:** 1874-02

**Authors:** J. E. Kelly

**Affiliations:** Surgeon to Jervis St. Hospital, and Lecturer on Anatomy and Physiology in the Ledwich School of Medicine, etc.


*Bloodless Surgery of the Extremities.* By J. E. Kelly, L.R.C.S.D., Surgeon to Jervis St. Hospital, and Lecturer on Anatomy and Physiology in the Ledwich School of Medicine, etc.

Within the last fortnight I have applied Esmarch's method to four surgical cases. This coincidence induces me to detail some facts directly bearing upon the question of "Bloodless Surgery," together with a few points worthy of consideration.

Whether we concede to Esmarch the honor of an invention, or merely the adaptation of a rarely applied and undeveloped principle, is of little moment to those who derive most benefit from an expedient which we must regard as constituting one of the most striking advances in modern surgery. Simplicity and easy application are perhaps the strongest recommendations of a method, the novelty of which will be my excuse for briefly describing it. A strong elastic bandage is applied in the ordinary manner to the extremity, firmly and slowly; a piece of india-rubber tubing of the diameter of the thumb is wound very tightly round the limb immediately above the margin of the bandage, which is then removed. The preparations are complete, and the limb is perfectly bloodless until the tubing is removed. The facility of operating under these circumstances can hardly be realized. The structures have a semi-transparency resembling that resulting from preservation in turpentine or glycerine, and, as may be expected, they are picked out and distinguished with remarkable facility. The operations upon which Esmarch's method confers the greatest advantage are those in which much blood is ordinarily lost, or those performed under circumstances which prevent the operator from providing efficient aid. Amongst the former, resections and explorations, either for foreign bodies or where preliminary investigations are judicious, may be specially noticed. These preliminary measures, so conservative in their aim and results, have often been frustrated by the danger or dread of the consequent hæmorrhage. Now we are enabled to pursue them bloodlessly. In provincial and in military practice a surgeon must frequently operate without skilled assistants, or perhaps without any. Here again the advantages of the method are indisputable.

Against the obvious advantages of the expedient we must in justice weigh some well-founded objections. While advocating, and in some instances practicing bloodless surgery, I regard it as being unnecessary in many cases, and in others as only to be used with great caution. In cases where collections of septic matter exist, Esmarch warns us against the danger of favoring by pressure its entrance into the circulation. The absence of bleeding points during the operation may cause us to neglect to secure some vessels which will subsequently be troublesome. This occurred to a suggestive extent in the second of the accompanying cases, but the hæmorrhage was quickly checked by the application of ice. As observed in the amputations performed in Guy's and other hospitals, the edges of the incisions may slough. I noticed this in my first case only, and my observations on it and the succeeding cases induce me to regard the sloughing as proportionate to and dependent on the duration of the pressure of the elastic bandage, and not to the bloodless condition of the tissues. In the first of the series, being anxious to do full justice to the method, I applied the bandage in the ward, before the removal of the patient to the operation theatre, with the view of making the limb perfectly exsanguineous. When the patient was chloroformed, the caoutchouc tubing was applied, and the bandage, after being on for about ten minutes, was removed. The results were perfect; so bloodless were the parts that in my subsequent operations I allowed the bandage to remain on but for a few minutes, and in my last, immediately after the limb was entirely enveloped, I applied the tubing and removed the bandage. This operation was completely bloodless, and along the line of the incision not a trace of sloughing could be observed; in some parts even there was an effort at union by adhesion, but the condition of the deeper structures would not admit of such a rapid closure. I am inclined to regard sloughing as unimportant in these cases, which are really benefited by the method. In amputations and other operations which can be completed in a few minutes, I cannot recognize the necessity for departing from the old and efficient method of controlling hæmorrhage by direct arterial compression. Hence, I question the importance of a contingency which can be avoided. In military and provincial practice, where, as already remarked, the surgeon must amputate without sufficient skilled assistants, he is free to select between the immediate danger of hæmorrhage and the remote disadvantage of sloughing of the flaps; observations are as yet deficient as to the constitutional effects of the expedient. The quantity of blood lost during operation is frequently a most desirable antiphlogistic. By Esmarch's method, for good or for evil, this is prevented, and severe inflammation may follow the operation; however, in such cases the surgeon can conveniently resort to phlebotomy, or other antiphlogistic treatment, as suggested by his judgment. Again, in disease or predisposition to congestion in any of the important viscera, as the lungs or brain, we may well hesitate before we throw the entire amount of blood contained in a limb, either temporarily or permanently, into the general circulation. We must also remember the possibility that this quantity of blood may embarrass the pulmonary circulation and aid in developing the fatal effects of the anæsthetic. These are points which can only be settled by experience, and they appear to me to be worthy of the consideration of the profession.

CASE I.—*Necrosis of the Entire Tibia.*—Thomas ——, æt. 17, disease of eight months duration. Commenced with violent pain about the tuberosity of the tibia. His leg became flexed, and an abscess formed which pointed and burst about the tenth day. A series of these occurred along the front of the leg. Admitted November 10th, with symptoms indicating necrosis of the entire shaft of the bone. Operation on November 15th. Commenced by an incision reaching nearly from the knee to the ankle. The new osseous case was three-quarters of an inch thick, and was opened with a chisel, at the expense of much labor, to the same extent as the primary incision. The bloodlessness of the operation may be estimated by the fact that when the sequestra were removed the cavity did not contain a drop of blood. The case is progressing most favorably, and the cavity is lined by healthy granulations. The edges of the incision sloughed in their entire extent, but only to a limited degree.

CASE II.—*Necrosis of Lower End of Tibia.*—Joseph ——, æt. 15, disease of two years duration. Commenced by a pain in the ankle, and an abscess formed above the articulation. The bone became expanded at its lower third, and a succession of abscesses formed on the anterior and inner surface of the leg. Admitted October 29th, with symptoms of necrosis of the lower end of the tibia. After some general treatment the operation was performed on November 13th. A portion of expanded bone was chiseled out, and a few small sequestra were removed from the centre of the mass. This case also progresses favorably. Some smart hæmorrhage occurred about two hours after operation, but it was easily suppressed by the application of ice.

CASE III.—*Necrosis of the Shaft of the Humerus.*—Margaret ——, æt. 15, disease of eight months duration. Gives the history of rheumetic fever, for which she received hospital treatment; abscesses formed at various points along the arm; admitted November 20th, and operation on the 25th of November. A long incision on the outer side of the arm, and the new osseous case, which was badly developed, was cleared of the sequestra. The special interest in this operation is owing to the application of the tubing above the arm, or round the clavicle and the neck of the scapula. Notwithstanding the protection which the vessels receive from the bones in the neighborhood of the last stage of the subclavian artery, this operation was as bloodless as any of the preceding ones.

CASE IV.—*Anchylosis of Elbow Joint; Resection.*—Anne ——, æt. 23, disease of four years duration, resulting from an injury to the joint. Admitted November 28th. Operation, sub-periosteal (November 29th), performed by means of an external incision. The osseous mass was cut through with a forceps, the end of the bone turned out and sawn off. The case progresses most favorably. The wound was dressed before the tubing was removed, and during, or after the operation, or at any time, not a single drop of blood was visible.—*Medical Press and Circular.*

